# Working Memory for Sequences of Temporal Durations Reveals a Volatile Single-Item Store

**DOI:** 10.3389/fpsyg.2016.01655

**Published:** 2016-10-26

**Authors:** Sanjay G. Manohar, Masud Husain

**Affiliations:** Nuffield Department of Clinical Neurosciences, University of OxfordOxford, UK

**Keywords:** working memory, attention, duration, serial position effect, focus of attention

## Abstract

When a sequence is held in working memory, different items are retained with differing fidelity. Here we ask whether a sequence of brief time intervals that must be remembered show recency effects, similar to those observed in verbal and visuospatial working memory. It has been suggested that prioritizing some items over others can be accounted for by a “focus of attention,” maintaining some items in a privileged state. We therefore also investigated whether such benefits are vulnerable to disruption by attention or expectation. Participants listened to sequences of one to five tones, of varying durations (200 ms to 2 s). Subsequently, the length of one of the tones in the sequence had to be reproduced by holding a key. The discrepancy between the reproduced and actual durations quantified the fidelity of memory for auditory durations. Recall precision decreased with the number of items that had to be remembered, and was better for the first and last items of sequences, in line with set-size and serial position effects seen in other modalities. To test whether *attentional filtering* demands might impair performance, an irrelevant variation in pitch was introduced in some blocks of trials. In those blocks, memory precision was worse for sequences that consisted of only one item, i.e., the smallest memory set-size. Thus, when irrelevant information was present, the benefit of having only one item in memory is attenuated. Finally we examined whether *expectation* could interfere with memory. On half the trials, the number of items in the upcoming sequence was cued. When the number of items was known in advance, performance was paradoxically *worse* when the sequence consisted of only one item. Thus the benefit of having only one item to remember is stronger when it is unexpectedly the only item. Our results suggest that similar mechanisms are used to hold auditory time durations in working memory, as for visual or verbal stimuli. Further, solitary items were remembered better when more items were expected, but worse when irrelevant features were present. This suggests that the “privileged” state of one item in memory is particularly volatile and susceptible to interference.

## Introduction

When a series of items is held in working memory, not all items are held with equal fidelity. Items early in the sequence may be forgotten, whereas items at the very start of a sequence may be easier to find. The *final* item in a sequence may also be held in a more “active,” privileged or prioritized state (Allen et al., 2014; Hu et al., 2016). This is known as the “recency effect,” and has been shown to be volatile, susceptible to a number of attentional manipulations (Davelaar et al., 2005; Hu et al., 2014). It decays quickly (Postman and Phillips, 1965; LaRocque et al., 2014), may be selectively impaired by TMS or lesions to modality-specific cortex (Vallar and Papagno, 1986; Zokaei et al., 2014a), and may relate to earlier items being forgotten through retroactive interference (Kool et al., 2014). For these reasons, it has been postulated that the benefits enjoyed by the final item in a sequence arise because it remains in the *focus of attention*.

Recent studies of working memory have begun to use continuous recall measures, which allow the precision or fidelity with which items are stored to be quantified. Most of these studies have used visual working memory, measuring the precision of storing spatial locations, colors or orientations (Bays and Husain, 2008; Zhang and Luck, 2008). These paradigms require participants to reproduce their memory of a continuously variable feature, for example by adjusting a dial. The reported feature can then be compared to the veridical feature, providing a trial-wise, quantitative precision measure. Recently, these precision paradigms have been extended to auditory and vibrotactile frequencies, and similar effects have been demonstrated, indicating that features in various modalities may all be encoded in a similar way (Kumar et al., 2013; Joseph et al., 2015). In neural models of working memory, the ability to hold several continuous features in memory has been taken to suggest that the feature dimensions are encoded in a set of independent feature-tuned channels, which are activated upon encoding each feature for each item in memory (Compte et al., 2000; Wimmer et al., 2014).

Could a similar storage method be used to hold temporal durations in memory? Periods of time are abstract: durations do *not* traditionally form a parameterised space represented by cells in sensory transduction. Intervals of time might need to be explicitly extracted or inferred from other kinds of representation (Matthews and Meck, 2016). Durations are also unusual things to hold in short-term memory. Despite this, it appears that we do in fact possess working memory for durations (Teki and Griffiths, 2014). Indeed we are able to repeat rhythms that we hear, for example in music, poetry or speech (McAuley, 2010). But it is not clear that the same mechanisms would be involved, as those that subserve visual or verbal working memory. The presence of set-size, serial position, and attentional effects could provide evidence for commonality of mechanisms.

Human time perception has been most commonly studied with simple interval estimation, reproduction and comparison tasks (Grondin, 2010). A number of factors increase or decrease the perceived duration of an interval. Practice can lengthen perceived durations (Eisler, 1976), as can arousal (Wittmann, 2013), whereas aging shortens them (Baudouin et al., 2006b). *Attention* and *expectation* play particularly important roles in interval timing. Attentional loads shorten perceived durations while they are experienced (Brown, 1985, 1997; Block et al., 2010) but lengthen the reproduction of durations (Fortin and Breton, 1995; Baudouin et al., 2006a,b). Evidence from patients also implicates attention in timing, with patients reporting shorter and less accurate estimates of durations (Danckert et al., 2007). We therefore studied whether attentional demands might alter retention of durations in working memory, by introducing variation of an irrelevant feature.

Importantly, expectation also impacts on timing. The presence of distractors during a time judgment task can lengthen the subjective duration of a stimulus, but this effect only arises when the distractors are unexpected (Penney et al., 2014). Similarly, producing an interval that is interrupted by a pause late in the interval leads to overestimation; this effect persisted on trials when a break did not actually occur, but was expected to occur (Fortin and Massé, 2000). These results suggest that expectation of an upcoming event shortens perceived durations. In the present study we investigate whether simply expecting an event could enhance memory retention for durations.

We set out to test a direct analog of visual working memory experiments, in the time domain. In particular we asked, does memory for durations show similar set-size and serial position effects as visual working memory? Further, we enquired whether set-size and serial position effects are susceptible to manipulation of attention and expectation. We asked whether the need to filter irrelevant information, and the expectation of the end of a sequence, altered the recency effect. We hypothesized that any attentional benefits would be attenuated if irrelevant features were being ignored. Regarding temporal expectation, we predicted that the unexpected end of a sequence can confer a recency benefit, whereas if the ends of sequences were expected, this advantage would be lost.

## General Methods

**FIGURE 1:**
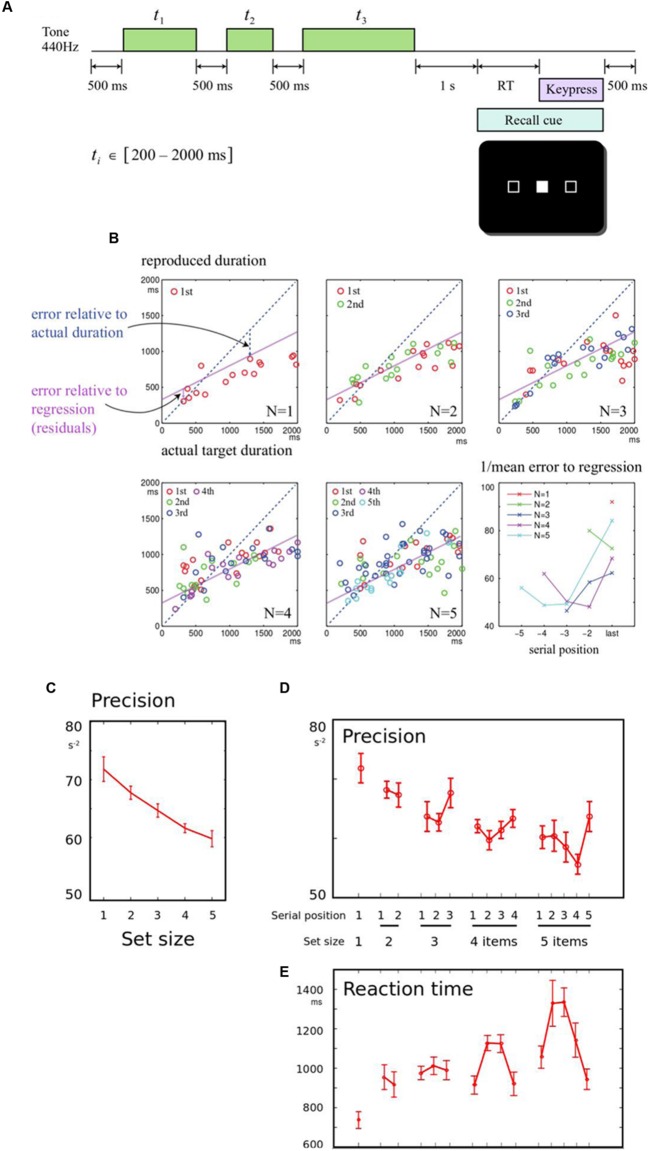
**(A)** Memory for durations task: In order to study how a series of durations are held in working memory, participants were asked to listen to a sequence of one to five tones. After a 1 s delay, they were cued to one of the tones by its serial position. They had to reproduce the duration of the cued tone by holding down a response key for a matching duration. The durations to be remembered were drawn from a uniform distribution between 200 and 2000 ms. **(B)** Example of results from a single participant: Panels correspond to sequence lengths 1–5 items. In each panel, the response durations of all trials are plotted, as a function of the corresponding target duration. The blue diagonal dotted line indicates perfect performance, where responses would be identical to the heard durations. The purple line indicates a linear regression fit to all the subject’s responses. The slope is flat and intercept is positive, indicating that short durations are overestimated and long durations are underestimated. Errors were calculated relative to the regression line. The final panel shows the precision (reciprocal of the root mean square error) calculated for each set-size and serial position, for this subject. Colors indicate different set-sizes, and the final item for each sequence length is aligned to the right. **(C)** Precision falls with longer sequences: When more durations needed to be remembered, the overall precision of reported durations was reduced. Data were collapsed across the serial positions, and the inverse mean error for each sequence length is shown. Error bars indicate within-subject error for the effect of set-size, across all participants. **(D)** Precision shows primacy and recency effects: The mean error was broken down by serial position, demonstrating an overall benefit for the last item in a sequence (recency effect). **(E)** Response times mirror memory precision: Responses were faster when fewer items had to be remembered. Serial effects were also observed, with faster responses for the first and last items in 4- or 5-item sequences (primacy and recency effects), as predicted by an information-accumulation model of response time.

Participants were instructed to listen to each sequence of tones, and remember the time each one lasted for. They were told that after a delay, they would see a signal indicating which of the items in the sequence they had to recall (probed by serial order), and that they had to press and hold a key to try and match that duration as precisely as they could (**Figure 1A**).

Participants sat in a dimly lit room viewing a CRT monitor at a distance of 40 cm from a chinrest. Tones were presented through a pair of stereo speakers, situated either side of the computer screen 50 cm in front of the subjects, at shoulder height. Tones comprised a sine wave at 440 Hz (Experiments 1 and 3). Each tone was modulated to taper linearly over the first and last 10 ms, to minimize transients. The durations to be remembered were selected from a uniform distribution between 200 and 2000 ms. Sequences of 1 to 5 durations (Experiment 1) or 1 to 4 durations (Experiments 2 and 3) were chosen, with proportionally more trials for higher set-sizes. This permitted each serial position in each set-size to be probed equally frequently. The tones were separated by a fixed 500 ms inter-stimulus interval. After the end of the final tone, there was a 1000 ms silent retention interval.

At the end of the retention interval, the computer screen displayed a cue indicating which item was to be recalled. This was done graphically by displaying a row of squares, each representing one of the items heard on the current trial, in sequential order from left to right. One square was filled in, indicating the item that had to be recalled. For example, if four tones were heard, there would be four boxes, and to indicate that the first tone should be recalled, the left-most square was filled in white, whereas the other three were hollow frames.

Participants then pressed and released the key, to indicate their memory of the duration of the indicated tone. After the response, an inter-trial interval of 500 ms followed, and the next trial began. No feedback was provided.

In all experiments, 10 practice trials were performed, and participants were debriefed to check they understood the task, before the experiment began.

## Experiment 1: Working Memory For Durations

### Methods

Experiment 1 required participants to remember 1- to 5-item sequences, and each serial position in the sequence was probed equally often. This gave 15 trial types, with more 5-item trials than 1-item trials. There were 60 trials per block, in 4 blocks, separated by a 2-min break. 15 participants performed this experiment.

Participants were recruited from the UCL Psychology subject pool, and were aged 18–36 years (mean 26.5 years). All subjects gave informed written consent as approved by the UCL Research Ethics Committee.

### Results

Our primary measure was recall error. As expected, there was an overall linear relationship between the recalled duration and the corresponding presented duration, and this relationship showed systematic overestimation of shorter intervals, and underestimation of longer intervals (**Figure 1B**). This demonstrates a well-studied bias in interval reproduction, and in line with other studies we used a linear fit to model this bias (Jazayeri and Shadlen, 2010, 2015). This permitted us to measure error relative to each individual’s linear fit, as the residuals of the regression. Thus on each trial, the discrepancy relative to this fit could be calculated, that indicated the fidelity of memory recall. Memory fidelity, or *precision*, was quantified as the reciprocal of the root mean squared error, calculated for each condition for each subject.

First, the effect of set-size was examined, collapsing across serial positions. Increasing set-size strongly reduced precision [**Figure 1C**, *F*(4,56) = 9.53, *p* < 0.001]. To establish how set-size and serial position influenced recall, a one-way repeated measures ANOVA was first performed across all set-size and serial position conditions. For five set-sizes, this gave 15 conditions. The conditions differed significantly [**Figure 1D**, *F*(14,193) = 3.46, *p* < 0.001]. The primacy effect was not significant [ANOVA of first and second item in sequences length 2 to 5, *F*(1,98) = 0.52, *p* > 0.05], but there was a significant recency effect [last vs. penultimate item in sequences length 2 to 5, *F*(1,98) = 6.64, *p* = 0.012].

A further linear regression within each condition produced similar results (Supplementary Figure S1), confirming that set-size and serial position effects were truly due to precision, rather than systematic bias. All results were also robust to normalizing by logarithmic transformations of the times (Supplementary Materials).

*Reaction times* (RT) were measured from the probe onset (appearance of filled box) until the button was initially depressed (initiation of the production interval). This interval therefore represents the time taken to identify the probed item, bring its duration to mind, and prepare a response. RT tended to be greater whenever precision was lower, and exhibited significant set-size effects (**Figure 1E**). RT was also faster for both the first and last items of a sequence, exhibiting both recency and primacy [*F*(1,98) = 7.22, *p* = 0.009 and *F*(1,98) = 7.60, *p* = 0.007]. The findings are in keeping with information-accumulation models of retrieval from memory that have been proposed in visual working memory (Pearson et al., 2014; Schneegans and Bays, 2016).

## Experiment 2: Effect Of Variation On An Irrelevant Feature Dimension

### Methods

We next asked whether the presence of an irrelevant feature would alter memory fidelity, as a function of set-size or serial position. Variation in this additional feature might invoke attentional filtering, and thus impair memory performance specifically for items that rely on attention.

**FIGURE 2:**
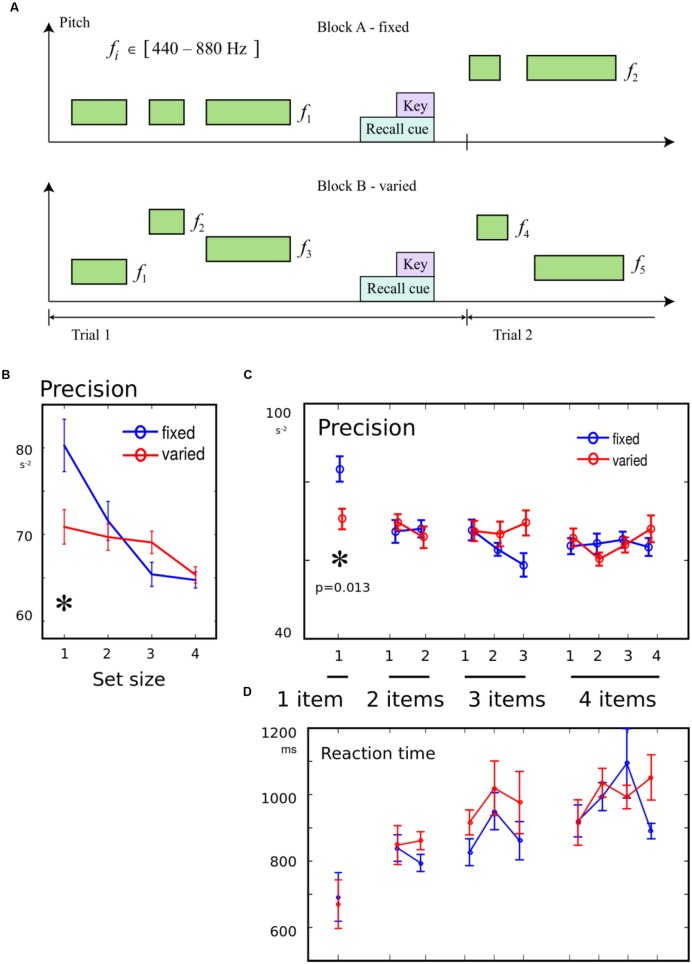
**(A)** Variation in pitch was an irrelevant feature: To examine whether the introduction of an irrelevant variation in pitch would worsen recall, two of the four blocks had the same pitch for all tones in each trial (“fixed” condition). In the remaining blocks, each tone on every trial had a randomly chosen pitch (“variable” condition). Between 1 and 4 tones were presented on each trial. **(B)** Precision for a single tone was worse in variable block: When only one item had to be remembered, memory precision was worse in blocks where pitches varied from trial to trial, compared to blocks when they were constant. There was no effect of variability when multiple items had to be remembered. **(C)** No effect of variability on serial position effects: The trials were broken down according to serial position in the sequence. Although there was an interaction of variability with memory condition, the only effect of variability was seen for the one-tone condition. **(D)** No effect of variability on reaction time (RT): There were no differences in the time to initiate a response, between variable blocks and fixed blocks.

In experiment 2, the pitch of the tone was randomly chosen between 440 and 880 Hz. It was emphasized to participants that the pitch was irrelevant, and that only duration had to be remembered. In some blocks, the pitch of *each tone* in a trial was varied randomly. In other blocks, the pitch of tones within a trial was kept constant, but randomly selected for each trial (**Figure 2A**). There were 1 to 4 items in each sequence, and different serial positions were probed on each trial. There were thus 10 combinations of set-size and probe position, with 9 repeats in each block giving 90 trials in each of four blocks. The two block-wise conditions, variable vs. constant pitches, were counterbalanced in order across subjects, such that eight participants performed blocks in the order “ABBA,” and eight in the order “BAAB.” For one participant, who did the constant block first, two blocks of data were lost, so their data were discarded, giving a total of 15 participants.

### Results

Precision was compared with a 2-way ANOVA, with factor 1 distinguishing the 10 possible combinations of set-size and probe, and factor 2 indicating the block type, i.e., the presence or absence of variation in the irrelevant feature. An interaction was observed between the probed item and presence of variation [*F*(9,266) = 2.16, *p* = 0.025], in addition to a main effect of item [*F*(9,266) = 2.56, *p* = 0.008], with no main effect of variability [*F*(1,266) < 0.1]. This interaction suggests that attentional filtering had selective effects on some memory conditions (**Figures 2B,C**). *Post hoc* tests revealed a that the interaction was driven by variation impairing recall specifically in the 1-item condition [*t*(14) = 2.83, *p* = 0.013], but no effects of variation were observed for any serial position for the other set-sizes. Therefore, only when a single duration had to be remembered, was there an effect of expecting variability in the current block. The same results were obtained when using separate regressions for each condition, set-size, and serial position. This indicates that the filtering effect was not due to a change in bias (Supplementary Materials). The pairwise tests were robust to normalization by log transform and non-parametric *U*-test.

There was no effect of variability on the primacy or recency effect, as quantified by interactions with the difference between the first two or last two items in 2- to 4- item sequences (both *F* < 1.26, *p* > 0.05).

Reaction times showed strong set-size effects as before (**Figure 2D**). However, there was no main effect of variability, no interaction with primacy [*F*(1,154) = 0.22], and a trend for variability to reduce the RT recency effect [*F*(1,154) = 3.27, *p* = 0.073].

Incidentally we noted that higher pitched tones were perceived as 1% longer in keeping with greater subjective intensity (Goldstone and Lhamon, 1974), but this effect did not interact with variability in our study (Supplementary Materials).

## Experiment 3: Effect Of Expecting A Sequence’s Length

### Methods

**FIGURE 3:**
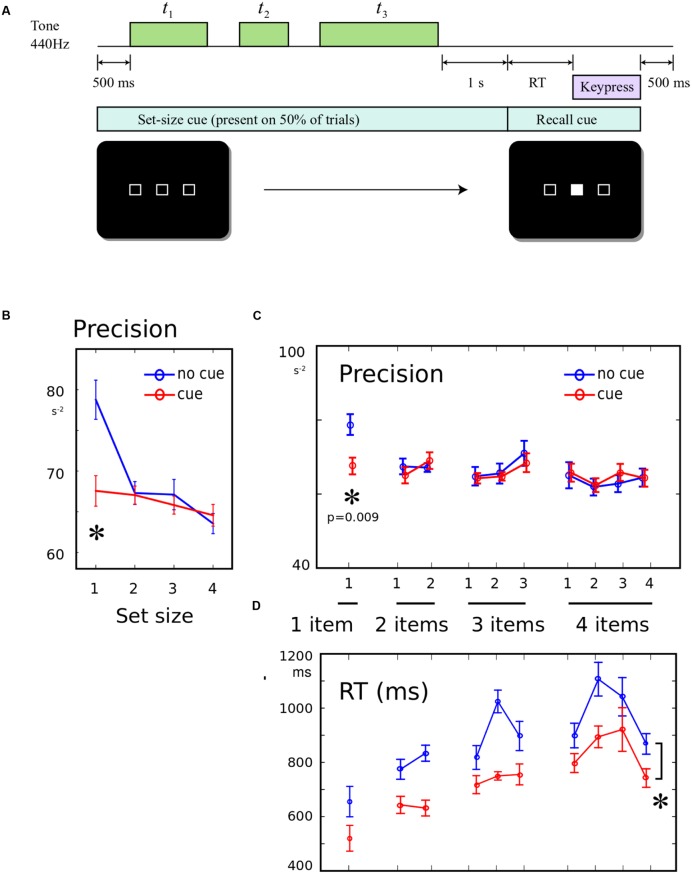
**(A)** Informing participants about the number of upcoming tones: 500 ms prior to the start of each trial, a screen was presented. On half the trials, this screen comprised a row of empty boxes, with the number of boxes corresponding to the number of tones that will be presented on this trial. The cue remained on-screen until the recall cue. On the remainder of trials, a cross was displayed instead, giving no information about the number of tones. **(B)** Precision for a single memory item was worse when number of items is known: The recall precision was significantly lower when a single tone was presented, if it was *expected* that the tone would be single, compared to when it was unexpectedly single. **(C)** No effect of pre-cueing set-size on other conditions: There was no worsening or improvement in recall when the number of items was known in advance, and there was no change in the shape of the serial position curve. **(D)** Response times faster when set-size was known in advance: On cued trials, RT was significantly shorter (main effect of cue). This did not interact with serial position.

In experiment 3, half the trials began with a cue screen lasting 500 ms, and the other half of trials began with a cross at the screen center. The cue screen consisted of a horizontal set of empty boxes, with the number of boxes indicating the number of tones that would be presented on the upcoming trial (**Figure 3A**). After the cue, the tones were presented and probed as in Experiments 1 and 2. There were 90 trials in four blocks, with all conditions interleaved. Fourteen participants performed the experiment, but one did not complete the task, leaving 13 datasets.

### Results

Precision was compared using a 2-way ANOVA, with factor 1 distinguishing the 10 possible set-size/probe conditions, and factor 2 indicating whether the set-size cue was present or absent. There was a main effect of item probed [**Figure 3B**, *F*(9,228) = 2.08, *p* = 0.032], no main effect of cue presence [*F*(1,228) = 1.54, *p* > 0.05], but a significant interaction between item and cue [*F*(9,228) = 2.00, *p* = 0.040]. This interaction was driven by a significant cue effect only for the 1-item condition [**Figure 3C**, *post hoc t*-test, *t*(12) = 4.04; *p* = 0.009], with no significant differences for any serial position in any other set-size. There was no effect of cue upon primacy or recency (both *F* < 0.23). The same effects were found when condition-wise regression was used to calculate the precision (Supplementary Materials), and the effect was robust to log-transform normalization and non-parametric *U*-tests.

Reaction times was significantly greater when no cue was presented [**Figure 3D**, *F*(1,228) = 36, *p* < 0.001], with no interaction with item [*F*(9,228) = 1.02, *p* > 0.05]. This indicated that expecting the end of the sequence improved the speed of responding generally. This confirms that cueing did indeed have the anticipated effect of improving expectation of when the probe would occur – but this was in sharp contrast to the above findings that precision was unchanged or *worse* with the cue.

## Discussion

This study asked whether memory for a sequence short auditory durations follows well-known laws associated with working memory in other modalities. The results confirm the existence of set-size and serial position effects that are in line with other modalities (**Figure 1D**). We then asked whether attention and expectation could modulate memory for durations. When a variable irrelevant feature was introduced into the sequences, memory for single items was worse, suggesting that the high performance normally observed for single items may be susceptible to attentional disruption (**Figure 2C**). When the number of items was expected, we found a similar disruption of the ability to recall a single item in memory. Thus single items are best remembered when they are unexpectedly the only item (**Figure 3C**). The benefit of having to remember one item, rather than a sequence is thus disrupted by the presence of irrelevant information, but enhanced when more items in the sequence are expected.

One possible interpretation of these findings is that both filtering and expectation interfere with a common aspect of the maintenance of singular items. Could the benefits for single items be mediated by an attentional focus, conferring more mnemonic resources on an item that is isolated? In attentional focus models, some items in memory are held in a “privileged” state (Oberauer, 2002; Cowan, 2011). If multiple items are held in working memory, not all items are recalled with the same accuracy, and some of the differences between items may arise because of a privileged or attended state conferred to one item (Lewis-Peacock et al., 2012; Postle, 2015). The benefit can be transferred among items (Zokaei et al., 2014b), and may explain the susceptibility of recency effects to attentional manipulations (Öztekin et al., 2010; Morrison et al., 2014; Souza et al., 2016). Our findings add weight to suggestions that working memory may contain one high-resolution but volatile representation. However, it is notable that we did *not* find attentional disruptions for the last item of longer sequences. This suggests that the recency effect might not always be susceptible to attentional load or expectation.

There may be other, more complex reasons for the disruption by expectation and filtering. Eye movements may distort time perception (Morrone et al., 2005; Burr et al., 2010) and thus the cue preceding the stimulus might alter time perception. Another possibility is that expectation of an event (e.g., further items that might be presented) can *increase* perceived durations (Fortin and Massé, 2000; Penney et al., 2014). Alternatively, a dual-task effect might occur in the filtering condition, which is known to *shorten* perceived durations of stimuli (Block et al., 2010). In Experiment 3 pitch differences between notes may also increase the perceived duration of gaps between tones (Crowder and Neath, 1995; Lake et al., 2014).

However, all these effects would be expected to lead to distortions of perception and thus *systematic biases* to over- or under-estimate the duration. In contrast, our results suggested no bias but an increase in error, i.e., the *variability* of responses around the same fixed duration. This might suggest that the measured effects occur at encoding or storage, rather than being perceptual biases. Could focusing attention explain the results of experiments 2 and 3?

An important property of attention is its refractoriness, as characterized by attentional blink or inhibition of return. These phenomena impose temporal capacity limits on deployment of attention to sequentially presented items. Could the set-size cue itself capture attention in experiment 3, and impair encoding of the subsequent tone? We think this is a less likely explanation, because the attentional blink tends to arise between 150 and 450 ms after a stimulus (Shapiro et al., 1997), whereas the gaps in our task were 500 ms. Further, this might also be expected to slow down RTs, whereas we in fact observed faster RTs. We suggest an alternative explanation: when the recall cue is expected after the item, the preparation of the response begins as soon as the tone ends. Note that such immediate response preparation cannot occur in any other type of trial, because either the item-to-be-probed is unknown, or the end of the sequence is not expected. Early response preparation could be the factor that leads to disruption of duration memory.

The present study is one of few that examine working memory for durations that are not intrinsically rhythmic. Most studies that investigate memory for multiple durations test our ability to discriminate rhythms, i.e., sequences of durations that are integer multiples of a discrete, quantised beat (Penhune et al., 1998; Teki et al., 2011; Grahn, 2012b). Such studies do demonstrate limitations in the number of durations that can be remembered, but give no indication of the precision with which each duration is remembered. Rhythm discrimination may in fact predispose subjects to use discrete categorical strategies for representing time, whereas for non-rhythmic time sequences, different neural mechanisms are thought to be recruited (Grahn and Brett, 2007; Grube et al., 2010; Joseph et al., 2016).

It is possible that in our task, durations could either be encoded individually, as absolute time intervals, or as relative times approximating a rhythmic structure. The present study is not able to distinguish these two possibilities. Rhythm perception involuntarily leads to complex changes in perceived intensity and timing, which vary according to expertise (Povel and Essens, 1985). Perceiving rhythm also leads to phase-dependent facilitation for many aspects of auditory perception and cognition (McAuley, 2010; Grahn, 2012a). Expecting further items in a sequence (Experiment 3) could potentially promote adoption of a rhythmic strategy, and this strategic change might drive the improvements observed with expectation. Rhythm-perceptual effects may be overlaid upon working memory effects, and could lead to more efficient storage of intervals at the expense of precision (Jones and Ralston, 1991; Large, 2002), similar to “lossy compression” or configural effects observed in visual memory (Alvarez, 2011). Further study would be required to directly measure the effect of rhythm-based encoding on duration memory.

We cannot exclude that our findings might be specific to the auditory modality, rather than representing a general effect in temporal cognition. The duration of auditory stimuli are generally reproduced more precisely than visual stimuli, with sounds being perceived as lasting around 20% longer than lights of matched duration (Goldstone and Lhamon, 1974; Wearden et al., 2006; McAuley and Henry, 2010). As we were primarily interested in the precision of memory, we used auditory stimuli for this experiment. Moreover, we tested temporal memory using “filled durations” – i.e., a tone lasting for the desired duration, as opposed to a gap in a tone, or the interval between a pair of clicks delineating an “unfilled” interval. The use of tones minimizes bias caused by start and end markers themselves (Rammsayer and Leutner, 1996), and produces more precise interval reproduction than the duration of gaps, whose durations tend to be systematically underestimated (Wearden et al., 2007).

How might neurones encode time durations in memory? Single time intervals could be reproduced by gradually varying neural activity during the encoding period which, upon termination of the interval, determines the subsequent rate-of-rise of an accumulator (Jazayeri and Shadlen, 2015) – somewhat like a pendulum that swings back to the height it was released from. But in order to use such an arrangement for sequences of several durations, an elaborate orchestration of segregated neuronal populations would be required (Kleinman et al., 2016). One way of achieving this might be to harness existing domain-general working memory processes.

Our results do suggest a conserved pattern of storage for remembering many different kinds of information, even including short durations. But for this to occur, durations should be encoded by a similar mechanism that has been proposed for other sensory modalities – i.e., a channel-based place code. What is the evidence that time intervals might be encoded by duration-selective channels? First, adaptation effects can be observed when we repeatedly hear a fixed duration, and these cross-modal adaptation effects are highly redolent of those observed in visual orientation and spatial frequency channels (Aaen-Stockdale et al., 2010; Heron et al., 2012). Second, neurophysiology provides evidence for duration-selective channels, for example with channels of the order of 30 ms in the brain stem (Brand et al., 2000; Aubie et al., 2009), around 100 ms in primary auditory cortex (He et al., 1997), up to 400 ms in V1 there, and in prefrontal cortex units have been found that are selective for durations up to 4 s (Yumoto et al., 2011). These neural representations could provide a substrate for storing duration information in working memory. Further, if duration-selective channels of this kind operate similarly to classical visuospatial or auditory feature domains studied in working memory, then similar capacity limits should be evident. In line with this, holding more than one duration in memory reduces the precision with which they can be remembered, and pre-cueing one of several durations can selectively improve memory (Teki and Griffiths, 2014).

Alternative classes of neural model have been proposed to explain how a single interval might be reproduced. First, pacemaker-accumulator models postulate a signal occurring at a fixed average rate that is integrated by a counter, and then compared to some threshold (Treisman, 1963; Gibbon et al., 1984). Second, population clock models posit that neural ensembles transition through a sequence of states in a probabilistic manner to produce accurate timing (Buonomano and Laje, 2010). Third, coincidences of noisy cortical oscillations may be detected by striatal neurons, rendering them sensitive to “beats” that occur after a learned interval (Oprisan and Buhusi, 2014). However, none of these proposals can straightforwardly account for the ability to hold multiple durations in mind, as observed in the current task. Functional imaging findings suggest that sensorimotor thalamocortical-basal-ganglia pathways may subserve the more complex aspects of temporal cognition (Schubotz et al., 2000; Schubotz and von Cramon, 2001; Grahn, 2012a). Indeed working memory may itself be central in producing an interval, because some form of counter needs to be maintained online during the interval (Brown, 1997; Gu et al., 2015). Individuating items in working memory and interval timing might utilize the same temporal context cues, an idea supported by correlations between memory performance and temporal discrimination performance (Unsworth and Engle, 2005; Broadway and Engle, 2011). Interval timing and working memory might thus be two modes of operation of the same neural system (Gu et al., 2015).

In summary, we show that several temporal durations can be held in working memory at once, and they are subject to standard sequential working memory limits. We demonstrated that the memory of single auditory durations in memory is especially susceptible to manipulations of attention and expectation.

## Author Contributions

SM conceived, conducted, and analyzed data from the experiments. SM and MH wrote the manuscript.

## Conflict of Interest Statement

The authors declare that the research was conducted in the absence of any commercial or financial relationships that could be construed as a potential conflict of interest.
