# Supplementary material for: Working Memory for Sequences of Temporal Durations Reveals a Volatile Single-Item Store
Source: Front Psychol. 2016 Oct 26;7:1655. doi: 10.3389/fpsyg.2016.01655 (PMC5080358; doi:10.3389/fpsyg.2016.01655)
Supplement: Supplementary file 1 [file Data_Sheet_1.DOCX]

# Supplementary material

**Precision effects using condition-wise regression**

Participants might systematically over- or under-estimate durations under certain conditions. Removing such effects is desirable because biases do not necessarily indicate decay of memory; for example, a large but stable bias is compatible with a strong memory representation of the item in memory. To rule out that bias could account for the variation in precision across conditions, we examined errors relative to a regression line in each condition. A separate linear regression was performed for each set size, serial position, and (in Expts 2 and 3) broken down by filtering or expectation. This ensures that the precision measure calculated from the residuals is independent of perceptual bias.


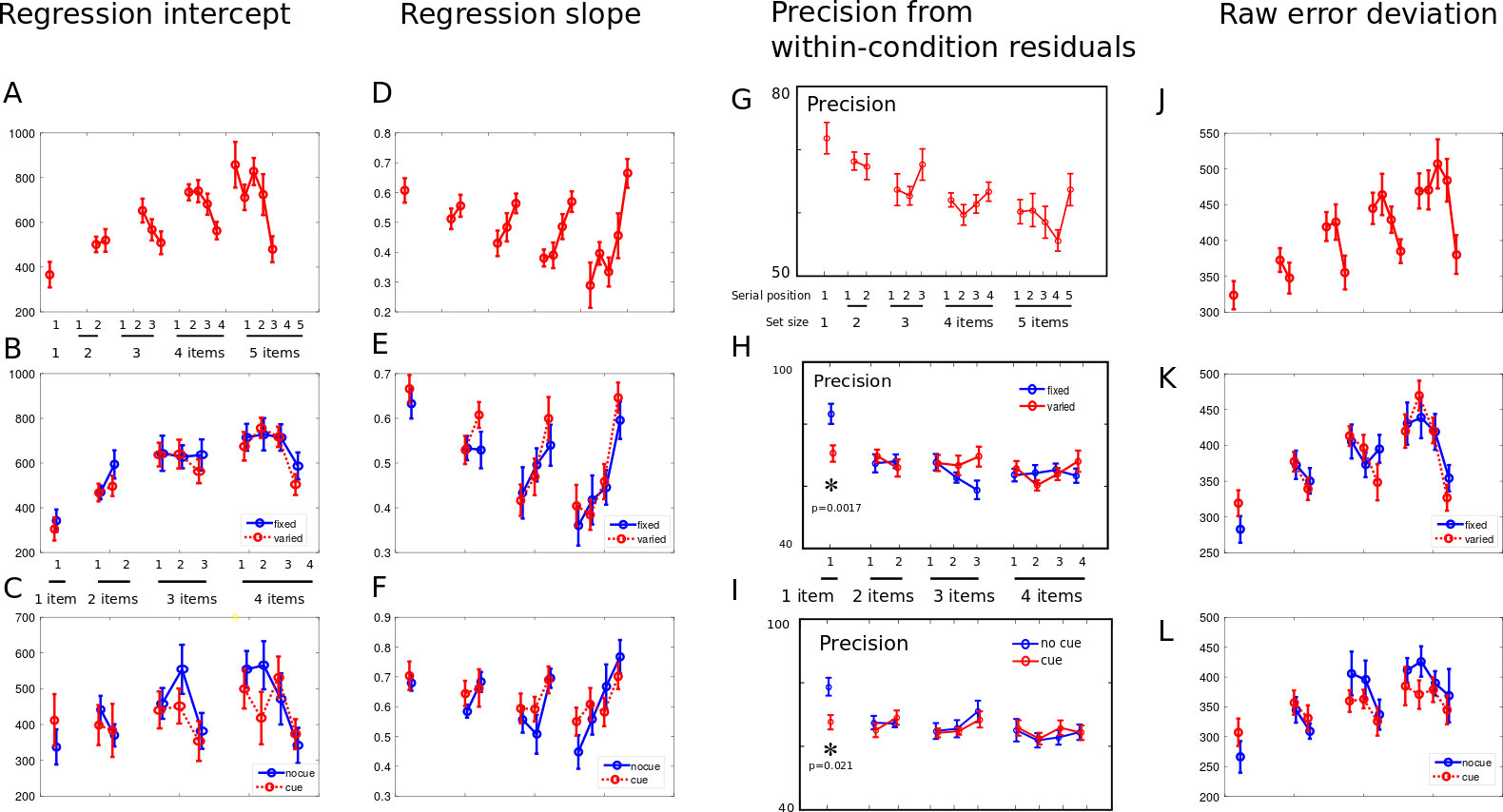


**Figure S1: Regression per condition separates out effects on biases and variation.**

Horizontal: first row is data from Expt 1, second row Expt 2, third row Expt 3.

A-F: Linear regression was performed on each subject and each condition, and results in an intercept and slope for each condition. The means and within-subject standard errors of these two parameter estimates across participants are plotted for each condition. There were significant effects of set size and serial position in all experiments. Importantly these parameters were not affected by the manipulations of Expts 2 and 3, indicating that the effects of filtering and expectation could not be explained in terms of bias to report intervals as longer or shorter.

G-H: To establish that the attentional effects were in fact due to variability, the residuals from the condition-wise regression were analysed just as in the primary analysis, by calculating the effective precision (1/standard deviation) for each condition. These are shown as subject means with standard error. The primary findings were reproduced, indicating that even after accounting for any differences in timimg bias, both filtering and expectation led to a precision difference when a single item was presented.

J-L: The raw error was calculated as the actual response duration minus the target duration on each trial. These values do not show significant effects of filtering or expectation, but would be expected to show a mixture of effects.

| Paired t-tests | 1 | 2:1 | 2:2 | 3:1 | 3:2 | 3:3 | 4:1 | 4:2 | 4:3 | 4:4 |
| --- | --- | --- | --- | --- | --- | --- | --- | --- | --- | --- |
| **Exp 2:** p= | 0.0017 | >0.05 |  |  |  |  |  |  |  |  |
| *t*(12) | 4.024 | 0.83 | -0.70 | 0.16 | 0.79 | 0.63 | 0.87 | 0.89 | 0.25 | 0.96 |
| **Exp 3:** p= | 0.021 | >0.05 |  |  |  |  |  |  |  |  |
| *t*(14) | 2.61 | .60 | .43 | -.14 | -1.14 | -2.09 | -.88 | 1.36 | -.19 | -1.04 |

**Table S1**: **Residuals from within-condition regression showed same effect as grand-regression residuals**

Pairwise comparisons of the effect of filtering (Exp2) and the effect of expectation (Exp3) were performed for each set-size and serial position. This analysis uses residuals calculated for regression within each condition, rather than the grand regression line. This means that the error values are not influenced by differences in biases between conditions. Two-tailed p-values are shown with paired-samples t-statistic below. Blank cells are all p>0.05.

**Analysis of Bias**

For each subject a single linear regression was performed for all trials across conditions, and residuals relative to this global regression were examined for each condition. The mean bias varied by condition (F(14,193)=3.16, p<0.001) such that items in longer sequences were remembered as longer, and items later in a sequence were remembered as longer (Fig S2).

**Tests on log-transformed times**

The calculation of linear regression residuals for a range of durations accounts for biases, but does not account for Weber-type effects, in which the variation in interval production scales up with the size of the interval (Zarco et al 2009; Acerbi et al 2012). Indeed the distributions of residuals did deviate from normality by the Kolmogorov-Smirnov test, applied to each participant’s error distribution individually (Fig. S4).

To test whether our ANOVA was robust to this deviation from normality, we re-calculated the linear regression using logarithms of time, i.e. regressing log(response duration) against log(target duration). This effectively makes the residuals *proportional* to the probed duration, rather than simply linearly deviates. These residuals were normally distributed.


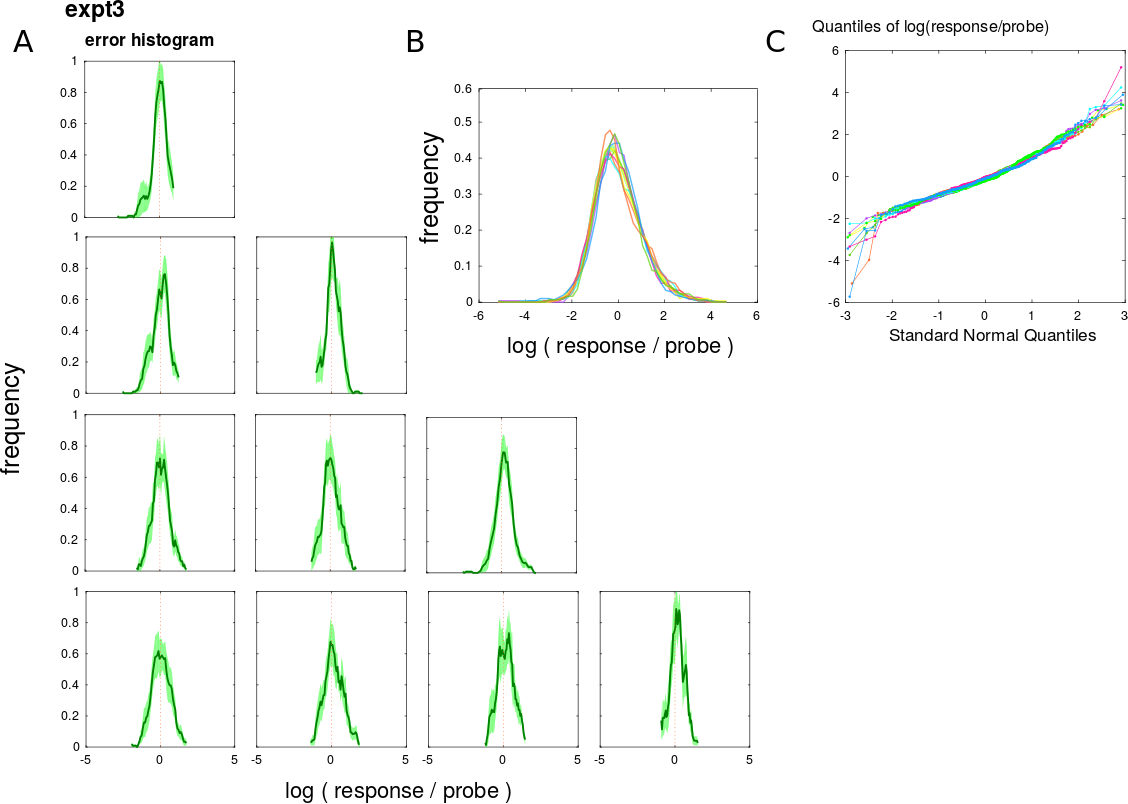


**Figure S2: Log proportional error is normally distributed.**

A: For each participant, set-size and serial position, a histogram of error values was constructed, and this histogram was averaged across participants to give the green curves. Each row is a set size, columns indicate serial position. A value of 0 indicates the probe is identical to the response.

B: Each participant’s log-proportional error histogram is shown in a different colour, with all conditions averaged. The curves for each subject did not deviate from normality according to the Kolmogorov-Smirnov statistic.

C: A quantile-quantile plot comparing each subject’s log-proportional error to the normal distribution. A straight line indicates normality; small deviations are noted at the extremes indicating a heavy-tailed distribution -- which is well recognised in working memory studies (Berg et al., 2012).

We then tested whether precision calculated from these residuals produced similar results. In particular, the effect of filtering and expectation upon the one-item condition revealed the same significant effects, albeit weaker (Expt 2 paired two-tailed t(14)=3.88, p=0.0017; Expt 3 t(12)=2.44 p=0.031), with no effects on other set-size and serial-position combinations (all t<1.61; p>0.05).

To check that our results were not driven by deviations from normality, we replicated all the pairwise comparisons using non-parametric tests (Mann Whitney signed-rank test) for experiments 2 and 3. The non-parametric statistics are reported in the following table S2, and can be seen to match the t-test results.

**Table S2: Nonparametric statistics replicated the effects of filtering and expectation in Expts 2 and 3**

| Signed Rank test | 1 | 2:1 | 2:2 | 3:1 | 3:2 | 3:3 | 4:1 | 4:2 | 4:3 | 4:4 |
| --- | --- | --- | --- | --- | --- | --- | --- | --- | --- | --- |
| **Exp 2:** p= | **0.010** | >0.05 | >0.05 | >0.05 | >0.05 | >0.05 | >0.05 | >0.05 | >0.05 | >0.05 |
| **Exp 3:** p= | **0.001** | >0.05 | >0.05 | >0.05 | >0.05 | >0.05 | >0.05 | >0.05 | >0.05 | >0.05 |

Since the distribution of residuals deviated from normality, nonparametric pairwise post-hoc comparisons were performed for each set size and serial position. The same pattern of results was observed, with significant effects on the single-item conditions.


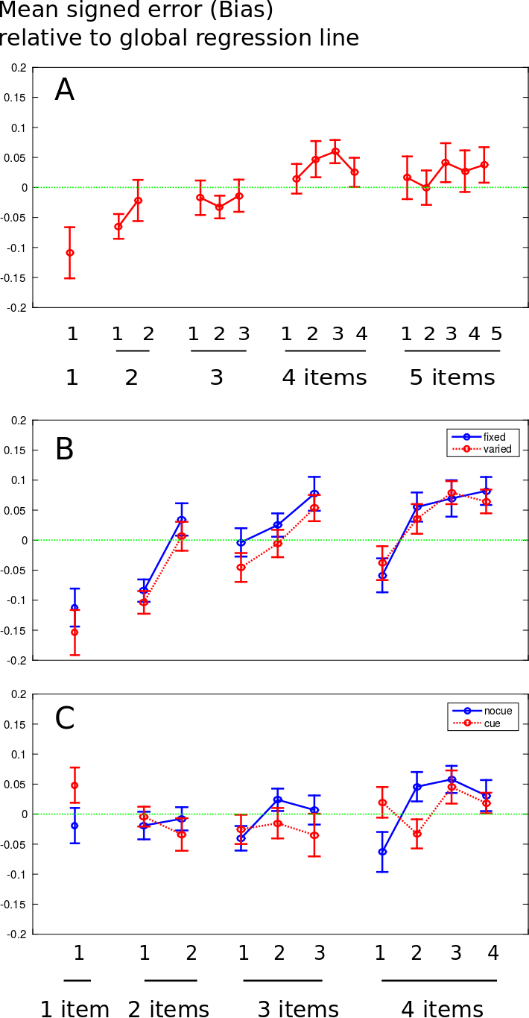


**Figure S3: Does bias vary according to condition?**

The relative bias in each condition was estimated, relative to the subject’s overall regression line. Values are mean signed residuals grouped by condition, with between-subjects standard error. **A**: experiment 1. Single items were reported as shorter relative to longer sequences. Items at the start of the seqeuence were reported as shorter relative to those at the end of a sequence. **B**: Experiment 2. There was no effect of introducing an irrelevant feature on the pattern of bias. **C**: Experiment 3. There was no effect of pre-cueing the number of items in the sequence. For this plot, the log-log correlation was used, but an identical pattern was observed with the linear correlation residuals.

To visualise more precisely the actual responses made in each condition, we binned trials according to the duration of the target, and calculated the mean response duration in that bin. This produces an empirical curve relating response to stimulus. The mean across subjects is shown in **Fig.** **S4**.

These plots corresponds to the single-subject data of the original figure 1B, but at the group level. Notably the effect of filtering and expectation upon the line is seen to be minimal.


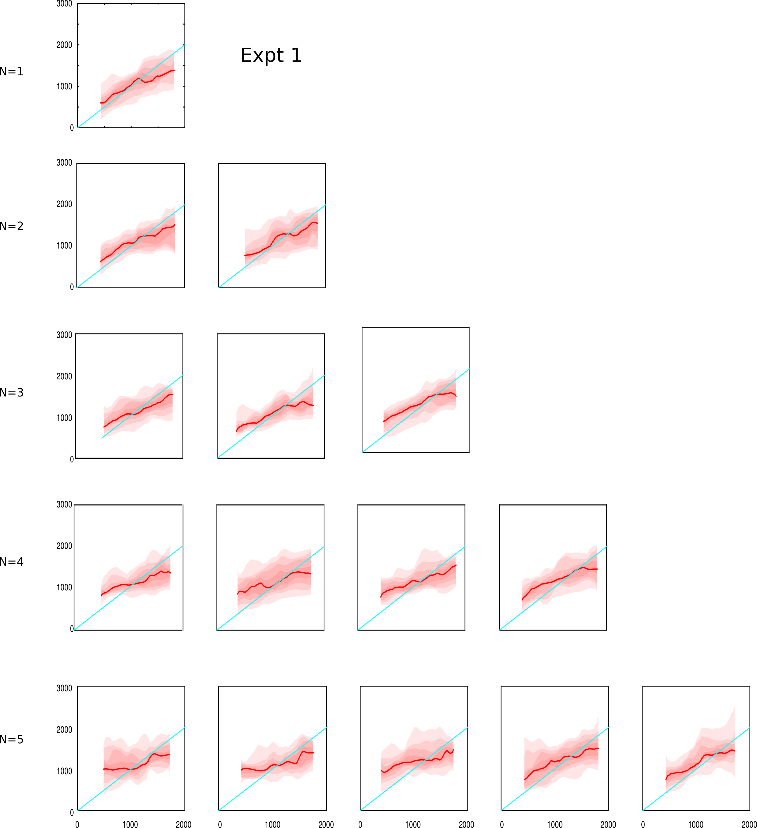


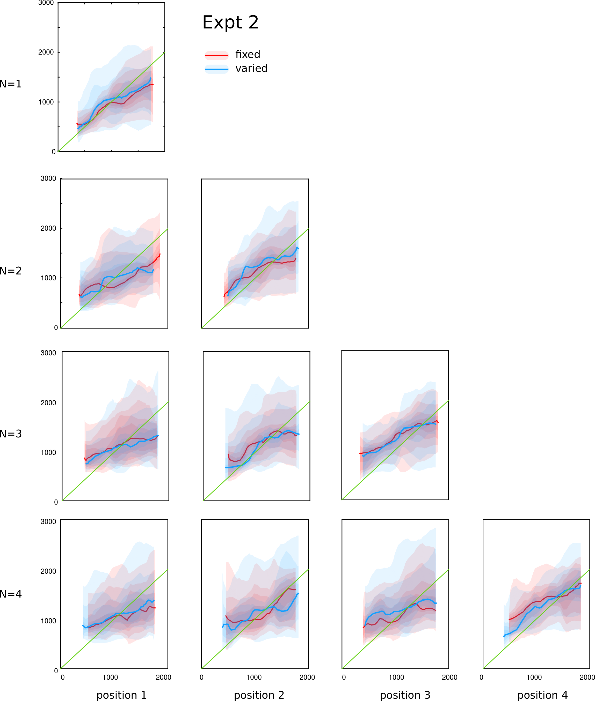

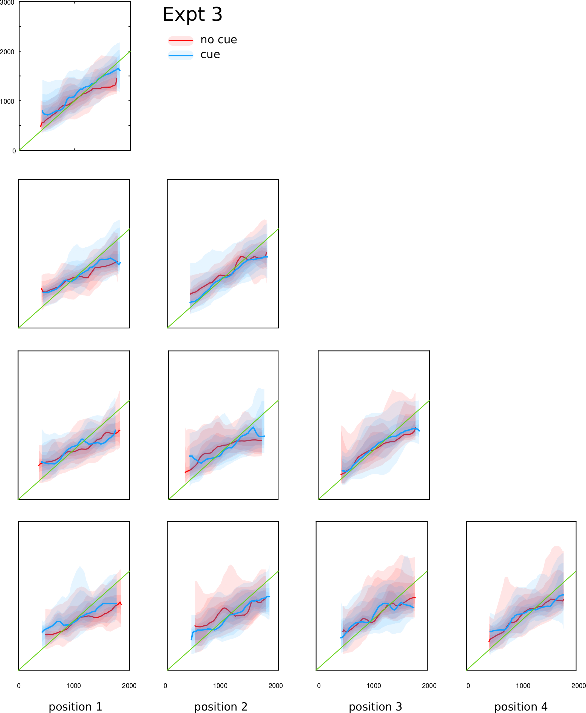


**Figure S4: Visualisation of empirical respose-vs-target curves**

For each subject and condition, trials were binned according to the target duration, using a sliding window 360 ms wide. The mean response duration in each bin was calculated. Line indicates mean across subjects and shading indicates every 0.125 quantile. The green line indicates a 1:1 ratio i.e. perfect responding. Flattening of the line occurs with regression to the mean due to memory degradation, or due to perceptual bias, or due to Bayesian estimation with a known prior.

**Does pitch influence response duration?**

The proportional error of the produced response relative to the probed duration for each trial was plotted against the pitch (**Fig. S5A**). There was no correlation in any condition (all r2<0.1, p>0.05) except for the first item in a sequence of 4. In this condition there was a weak correlation (r2=0.026, p=0.01 uncorrected for multiple comparisons) suggesting that higher pitched tones were remembered to be slightly longer in duration. However, slopes did not differ significantly between the conditions (1-way ANOVA F(9,130)=1.9, p>0.05).


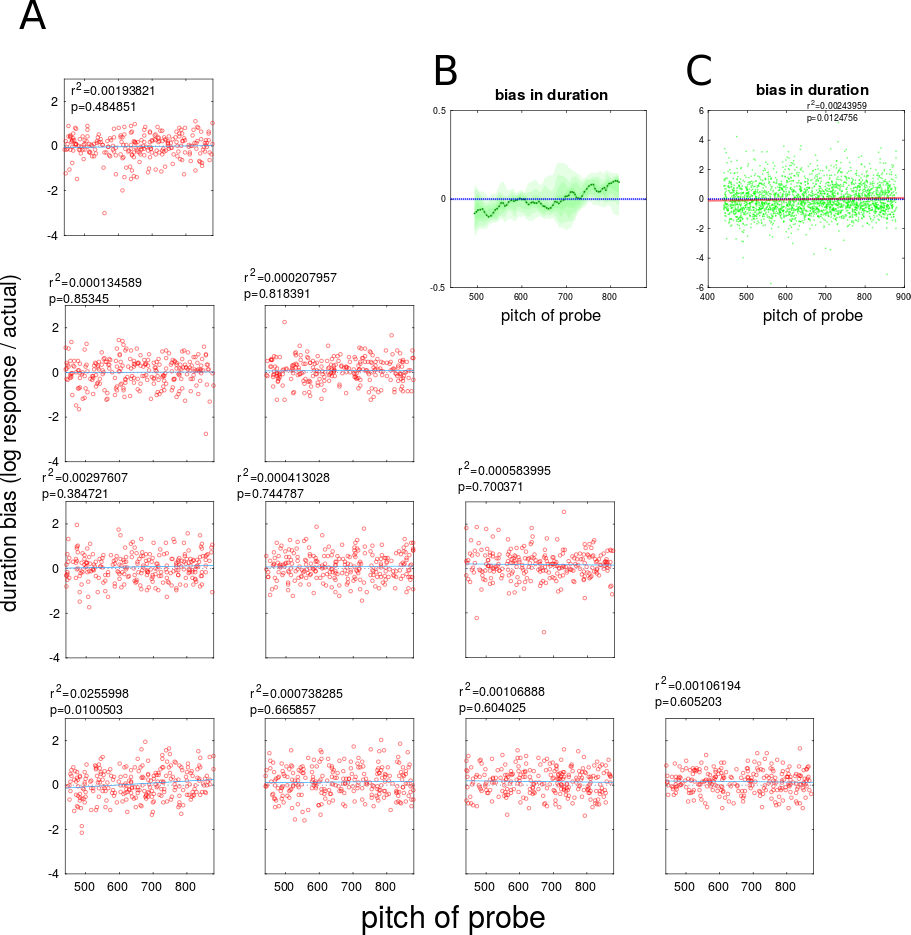


**Figure S5: effect of probe pitch on duration of response**

To investigate whether the pitch of the probe can alter the duration of the response, we performed a condition-wise linear regression of the log-proportional error, log(response / target), against probe tone frequency. A: graphs each show one combination of set-size and serial position, with rows indicating number of items, and columns as serial position. The zero line indicates perfect performance, and the blue line indicates linear fit. No significant relationship was found except in the first item in a 4-item sequence (lower left panel). B: Combining all trials, data was binned according to probe pitch and the mean response error in each bin was calculated (green line) with shading indicating each 0.15 quantile of error. C: The combined data across all trials indicated a small but significant effect with higher pitches perceived as longer.
